# Assessment of hand kinematics using inertial and magnetic sensors

**DOI:** 10.1186/1743-0003-11-70

**Published:** 2014-04-21

**Authors:** Henk G Kortier, Victor I Sluiter, Daniel Roetenberg, Peter H Veltink

**Affiliations:** 1 Institute for Biomedical Technology and Technical Medicine (MIRA), University of Twente, P.O. Box 217, 7500 AE Enschede, Netherlands

## Abstract

**Background:**

Assessment of hand kinematics is important when evaluating hand functioning. Major drawbacks of current sensing glove systems are lack of rotational observability in particular directions, labour intensive calibration methods which are sensitive to wear and lack of an absolute hand orientation estimate.

**Methods:**

We propose an ambulatory system using inertial sensors that can be placed on the hand, fingers and thumb. It allows a full 3D reconstruction of all finger and thumb joints as well as the absolute orientation of the hand. The system was experimentally evaluated for the static accuracy, dynamic range and repeatability.

**Results:**

The RMS position norm difference of the fingertip compared to an optical system was 5±0.5 mm (mean ± standard deviation) for flexion-extension and 12.4±3.0 mm for combined flexion-extension abduction-adduction movements of the index finger. The difference between index and thumb tips during a pinching movement was 6.5±2.1 mm. The dynamic range of the sensing system and filter was adequate to reconstruct full 80 degrees movements of the index finger performed at 116 times per minute, which was limited by the range of the gyroscope. Finally, the reliability study showed a mean range difference over five subjects of 1.1±0.4 degrees for a flat hand test and 1.8±0.6 degrees for a plastic mold clenching test, which is smaller than other reported data gloves.

**Conclusion:**

Compared to existing data gloves, this research showed that inertial and magnetic sensors are of interest for ambulatory analysis of the human hand and finger kinematics in terms of static accuracy, dynamic range and repeatability. It allows for estimation of multi-degree of freedom joint movements using low-cost sensors.

## Introduction

Analysis of hand kinematics is important in several application areas, such as rehabilitation, sports, ergonomics and animation industry. In particular, ambulatory tracking of the whole hand configuration is valuable for kinematic assessment under daily life conditions. This paper describes a new kinematic tracking system for the human hand which is based on inertial and magnetic sensors and offers various benefits compared to existing systems.

Current hand capturing systems can be divided in two categories, namely camera-based systems and datagloves.

Camera-based systems either use the contours of the hand or are guided by markers attached to the finger segments. The major drawback of camera based-systems is that the measurements to be performed are restricted to the volume in which the cameras are placed. In addition, occlusion of the hand-segments or markers result in a non-observable situation, inducing a poor estimate of the hand pose [1,2].

Datagloves form a large group of sensing devices that are worn on the hand. They differ in the way kinematic information is obtained. Two popular sensing methods are resistive-bend sensors and optical fiber sensors, with the latter one giving the highest accuracy (<1 deg), [3].

Disadvantages of both methods are related to sensor placement. Both measure the relative orientation of articulated segments by mounting the sensor across the joint of interest. This requires an accurate alignment of sensors with the particular joint. Often, re-calibration during utilisation is necessary to mitigate estimation errors due to sensor displacements.

A third sensing method used in datagloves is based on local magnetic actuation. Those sensors provide a high resolution without crossing finger joints. However, the cost of such a system rapidly increases as the degrees-of-freedom required increases. In addition, a magnetic actuator is required and manipulating ferromagnetic objects could interfere with the actuation signals [4,5]. An exception are passive magnetic systems, which are low cost and easy to wear [6,7]. However, they only allow to estimate a reduced set of kinematic finger variables.

A general disadvantage of datagloves is the lack of user customisation for individual subjects’ hands and obstruction of tactile sensing from the palmar surface of the hand. Often this inherently goes with mounting space required for embedding the sensors in clothing.

Inertial and Magnetic Measurement Systems (IMMS), containing inertial and magnetic sensors, have proven to be accurate in estimating body segment orientations without the need for external actuators or cameras [8]. The availability of Micro Electrical Mechanical Systems (MEMS) technology resulted in tiny and low-cost IMMS devices that can be implemented in textile clothing easily without impairing the freedom of movement and tactile sensation.

A glove system using accelerometers was presented in [9]. The system uses six dual axis accelerometers placed on the back of the hand and fingers. It was able to detect different static postures of the hand, which is useful for sign recognition. An extended version using triaxial accelerometers was presented, which was able to recognise more complex postures and simple gestures as well [10]. However heading observation was not examined and only a limited number of joints could be measured independently. Often, existing glove systems have been extended with a single IMMS placed on the back of the hand providing 3D orientation of the hand.

A glove instrumented with multiple IMMS’ has never been proposed to our knowledge. We propose a novel data glove that uses inertial combined with magnetic sensors placed on various hand and finger segments which is able to accurately assess full 3D hand and finger kinematics. Multiple extended Kalman filters (EKF) are designed to estimate the optimal orientation trajectories of hand and fingers. Change in hand position can be measured during short movement intervals.

In addition to presenting the instrumented glove, including sensor fusion methods, we evaluate the static accuracy, dynamic range and reproducibility of the system.

## Methods

The kinematics of each finger and thumb are treated individually and calculated using forward kinematics outlined in the next section. Subsequenly, four sections exploit an extended Kalman filter for the calculations of optimal relative finger, and absolute hand kinematics. Finally the experimental methods will be elucidated.

### Determination of phalangeal joint angles and finger tip position

**Figure 1:**
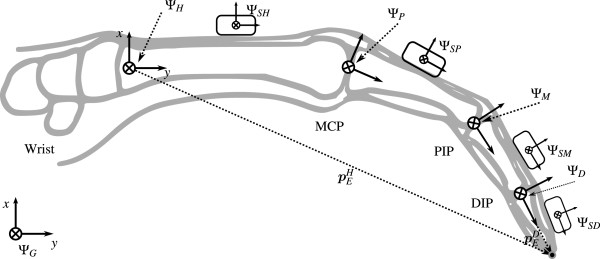
**Sagital view of the left index finger.** Given are the coordinate frame definitions for hand (**Ψ**_*H*_), proximal (**Ψ**_*P*_), medial (**Ψ**_*M*_), distal (**Ψ**_*D*_) segment, and corresponding joints: Meta Carpal Phalangeal (MCP), Proximal Inter Phalangeal (PIP) and Distal Inter Phalangeal (DIP). To all segments a triaxial gyroscope-accelerometer combination was attached. In addition the hand and distal segment include a magnetometer as well. The coordinate frame of various sensors is indicated with an S placed in front of the letter that indicates the segment. The position of the finger tip ***p***_*E*_ expressed in the hand frame **Ψ**_*H*_ can be calculated using the joint positions ***p***_*ij*_ and relative orientations R^*ij*^, were *i*,*j* are two connected segments. Figure modified from Wu et al. [11].

The articulated finger configuration can be modeled as a kinematic chain, originating from the hand coordinate frame **Ψ**_*H*_, see Figure 1. For the left hand this frame is defined by the *y*-axis pointing to the metacarpophalangeal (MCP) joint of the middle finger (distal), the *x*-axis pointing outwards with respect to the back of the palm (dorsal) and the *z*-axis is defined according the right-handed coordinate frame (radial). The proximal, medial and distal phalanges are modeled as rigid bodies of which the local coordinate frame is defined such that the *z*-axis is aligned with the functional flexion-extension axis (radial) of the joint and *x*-axis pointed dorsally. This definition is in accordance with the ISB [11] recommendations with positive angles for flexion (*z*-axis), abduction (*x*-axis) and pronation (*y*-axis).

The position of the finger tip $p_{E}^{H}$, expressed in the hand coordinate frame, see Figure 1, can be derived using forward kinematics:

(1) $$\left[\begin{array}{c} p_{E}^{H}\\ 1 \end{array}\right]=H^{\text{HP}}H^{\text{PM}}H^{\text{MD}}\left[\begin{array}{c} p_{E}^{D}\\ 1 \end{array}\right]=H^{\text{HD}}\left[\begin{array}{c} p_{E}^{D}\\ 1 \end{array}\right]$$

Where, the transformation between two consecutive bodies is expressed by H^*H* *P*^, H^*P* *M*^ and H^*M* *D*^. The superscript denotes the two coordinate frames of which the transformation is described; Hand (H), Proximal (P), Medial (M) and Distal (D). The total transformation H^*H* *D*^ is given by the product of each consecutive contribution:

(2) $$H^{\text{HD}}=\left[\begin{array}{cc} R(q^{\text{HD}}) & p_{D}^{H}\\ 0_{3}^{T} & 1 \end{array}\right]$$

where R(*q*^*H* *D*^) is the orientation of the distal phalanx with respect to the hand, and $p_{D}^{H}$ is the position of the distal frame expressed in the hand frame. The rotation matrix is defined by a unit quaternion, described in the Appendix, because they require a minimal set of parameters and have some appealing mathematical properties [12].

The relative orientation between two bodies can be obtained by solving the following differential equation [12]:

(3) $${\dot{q}}^{\text{ij}}=q^{\text{ij}}⊙\frac{1}{2}\omega_{\text{ij}}^{j}$$

where *q*^*i* *j*^ is the unit quaternion describing the orientation of frame **Ψ**_*j*_ with respect to frame **Ψ**_*i*_, ⊙ is the quaternion multiplication operator [12], and $\omega_{\text{ij}}^{j}$ is the angular velocity of body *j* with respect to **Ψ**_*i*_ expressed in frame **Ψ**_*j*_.

The relative angular velocity $\omega_{\text{ij}}^{j}$ is obtained by subtracting the absolute angular velocities of two articulated bodies. The angular velocity of a single body is measured using an 3D rate gyroscope, whose output ***y***_Ω_ can be modeled as:

(4) $$y_{\Omega }^{b}=\omega_{\text{Gb}}^{b}+b_{\Omega }^{b}+e_{\Omega }$$

where $\omega_{\text{Gb}}^{b}$ is the angular velocity of the body with respect to a global frame expressed in the body frame, $b_{\Omega }^{b}$ a slowly varying sensor bias and ***e***_Ω_ independent identically distributed (i.i.d.) white Gaussian noise. Subsequently, the relative angular velocity between two linked bodies (*i* and *j*) can be modeled as:

(5) $$\omega_{\text{ij}}^{j}=\left(y_{\Omega }^{j}-b_{\Omega }^{j}-e_{\Omega }^{j}\right)-R^{\text{ji}}\left(y_{\Omega }^{i}-b_{\Omega }^{i}-e_{\Omega }^{i}\right)$$

### Filter design: relative finger orientation

An extended Kalman filter structure is designed for optimal estimation of phalangeal orientations. The filter operates on the error of the actual state. This method has an excellent reputation in navigation purposes for airplanes and satellites [13] and, more recently, for MEMS based IMMS tracking as well [14-17]. It is advantageous to ordinary extended Kalman filtering because differences in estimated and true orientation is assumed to be much smaller than the actual orientation difference, which eventually result in a smaller linearization error. In addition, it is an appropriate method to circumvent the constraint in orientation descriptions. We will use the multiplicative error quaternion method [13], where the filter operates on the error quaternion which can be expressed as a non-constrained vector. Parameterization of the true quaternion *q*^*i* *j*^ by the nominal quaternion ${\bar{q}}^{\text{ij}}$ and error quaternion *δq* is given by:

(6) $$q^{\text{ij}}={\bar{q}}^{\text{ij}}⊙\delta q$$

**Figure 2:**
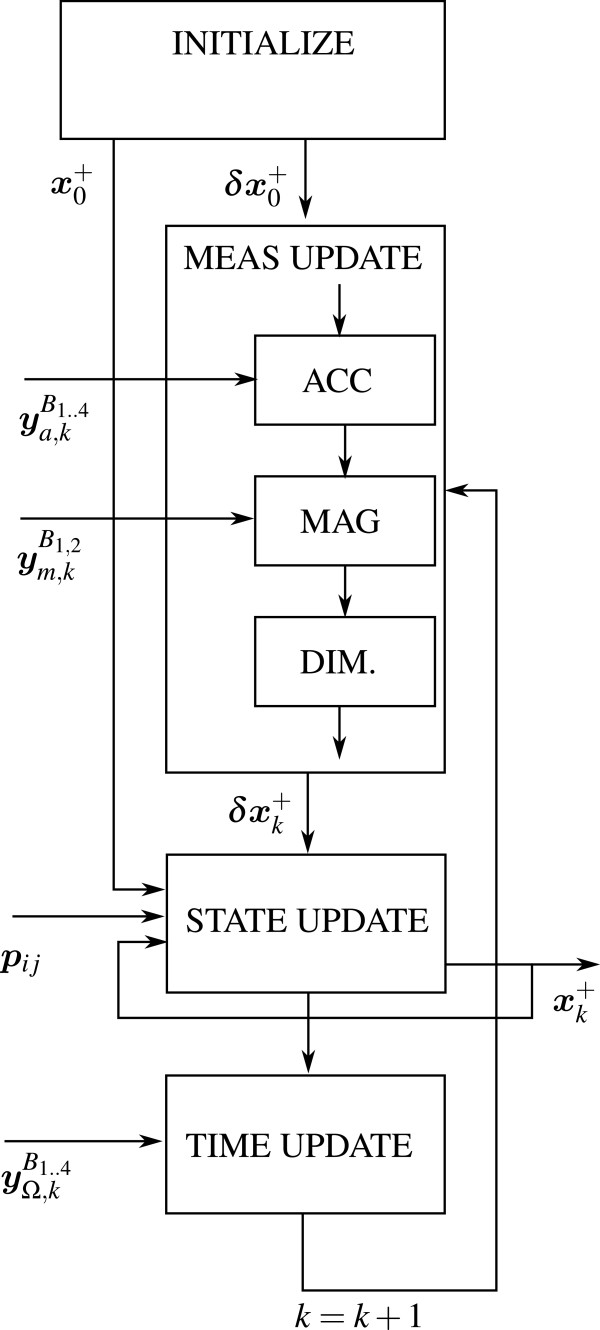
**Filter topology for each finger.** After initialization of both the state ***x*** and error state *δ****x***, a measurement update of error states according the measured acceleration and magnetometer signals (***y***_*a*_, ***y***_*m*_) and biomechanical dimensionaly information (DIM) can be performed. Thereafter states are updated, which are the relative orientations, finger tip positions and gyro bias estimates. Subsequently gyroscope information ***y***_Ω_ is used as an input to perform a update of the process (time update). Finally signals are propagated (*k*=*k*+1), and the described procedure is repeated.

Subsequently the error quaternion can be approximated using helical angles *δ* ***θ***^*i* *j*^:

(7) $$\delta q\approx {\left[\begin{array}{cc} 1 & \frac{1}{2}\delta \theta^{\text{ij}} \end{array}\right]}^{T}$$

where ***θ***^*i* *j*^ is the unit vector indicating a rotation axis and *δ* is the magnitude of the rotation around that axis. For each finger and thumb a single Kalman filter is deployed of which the structure is illustrated in Figure 2.

The filter uses a general state space model for dynamics ***x***_*k*+1_ and measurements ***y***_*k*_:

(8) $$\begin{array}{ll} x_{k+1} & =f(x_{k})+v\; \end{array}$$

(9) $$\begin{array}{ll} y_{k} & =h(x_{k})+e\; \end{array}$$

Where *f*(***x***_*k*_) and *h*(***x***_*k*_), denote the transition and measurement function respectively. Process and measurement noise contributions are given by ***v*** and ***e***. The state ***x*** is defined as:

(10) $$x={\left[\begin{array}{ccccc} p_{E}^{H} & q^{\text{HP}} & q^{\text{PM}} & q^{\text{MD}} & b_{\Omega ,l}^{b} \end{array}\right]}^{T}$$

where $p_{E}^{H}$ is the finger tip position, *q*^*i* *j*^ are the relative orientations between phalangeal segments. Because MEMS based rate gyroscopes have a low bias stability which would result in erroneous estimates of the orientation when the gyro output is integrated over long periods, gyro bias ($b_{\Omega ,l}^{b},l=1\mathrm{..}4$) has to be estimated over time, and therefore included in the state vector.

The filter operates on the error state which is defined as:

(11) $$\delta x={\left[\begin{array}{cccc} \delta \theta^{\text{HP}} & \delta \theta^{\text{PM}} & \delta \theta^{\text{MD}} & \delta b_{\Omega ,l}^{b} \end{array}\right]}^{T}$$

which include the error angles of various relative orientations and the error bias estimates of gyroscopes.

After state initialisation, the filter is fed with information from various sensors and performs each iteration one or multiple measurement updates, a state update and time update. We distinguish measurement updates from accelerometer and magnetometer, both denoted by ***y***_*v* *e* *c* *t* *o* *r*_ and biomechanical information, denoted by ***y***_*b* *i* *o* *m* *e* *c* *h*_.

During each measurement update step, the Kalman gain is calculated and the error state with its covariance are updated according [18]:

(12) $$\begin{array}{ll} K_{k}^{} & =P_{k}^{-}H_{k}^{T}{\left[H_{k}^{}P_{k}^{-}H_{k}^{T}+R_{k}\right]}^{-1}\; \end{array}$$

(13) $$\begin{array}{ll} P_{k}^{+} & =\left[I-K_{k}^{}H_{k}^{}\right]P_{k}^{-}\; \end{array}$$

(14) $$\begin{array}{ll} \delta {\hat{x}}_{k}^{+} & =\delta {\hat{x}}_{k}^{-}+K\left[\delta y-h(\delta x_{k}^{-})\right]\; \end{array}$$

Where, the minus and plus sign denote the a-priori and a-posteriori estimate respectively, *H* denote the linearized (or sensitivity) matrix of the measurement equation ***h*** (*δ* ***x***), *I* is an identity matrix, *R* is the measurement covariance corresponding to the measurement uncertainty and *δ* ***y*** the difference in estimated and measured sensory input.

During the state update, nominal states are updated accordingly to:

(15) $$\begin{array}{lll} q_{k}^{\text{ij}} & =\;{\bar{q}}_{k}^{\text{ij}}⊙\exp(\frac{1}{2}\delta \theta^{\text{ij}})\;\\ b_{k} & =\;{\bar{b}}_{k}+\delta b\; & \; \end{array}$$

where exp denotes the quaternion exponential, given in the Appendix. In addition, the tip position $p_{E}^{H}$ is updated according to equation 1.

Finally, during the time update, error states are set to zero and the corresponding covariance *P*, is propagated according the discretized process model *F* (described in the next section) and process noise *Q*.

(16) $$P_{k+1}=F_{d}P_{k}F_{d}^{T}+Q_{d}$$

### Relative orientation filter: process model

The estimated angular velocity is given by, see equation 5:

(17) $${\hat{\omega }}_{\text{ij}}^{j}=\left(y_{\Omega }^{j}-{\hat{b}}_{\Omega }^{j}\right)-{\hat{R}}^{\text{ji}}\left(y_{\Omega }^{i}-{\hat{b}}_{\Omega }^{i}\right)$$

The true orientation is derived using a rotation matrix approximation of equation 6 where the nominal orientation is given by the actual estimate:

(18) $$R^{\text{ij}}\approx {\hat{R}}^{\text{ij}}\left[I+{\left[\delta \theta^{\text{ij}}\right]}_{\times }\right]$$

where []_×_ denotes a skew-symmetric matrix. Finally, one can deduce the following equations to describe the error propagation process *F*_*d*_ (A detailed derivation of the equation is given in the Appendix):

(19) $$\begin{array}{ll} \delta {\dot{\theta }}^{\text{ij}} & =\;-{\left[{\hat{\omega }}_{\text{ij}}^{j}\right]}_{\times }\delta \theta^{\text{ij}}+\delta \omega_{\text{ij}}^{j}\; \end{array}$$

(20) $$\begin{array}{ll} {\dot{\delta b}}_{\Omega } & =\;e_{b}\; \end{array}$$

using equation 5, 17 and 18 yields the difference angular velocity:

(21) $$\begin{array}{lll} \delta \omega_{\text{ij}}^{j} & =\;\omega_{\text{ij}}^{j}-{\hat{\omega }}_{\text{ij}}^{j}\;\\ & =\;{\hat{R}}^{\text{ji}}{\left[y_{\Omega }^{i}-b_{\Omega }^{i}\right]}_{\times }\delta \theta^{\text{ji}}\; & \;\\ & \;+{\hat{R}}^{\text{ji}}\delta b_{\Omega }^{i}-\delta b_{\Omega }^{j}-e_{\Omega }^{j}+R^{\text{ji}}e_{\Omega }^{i}\; & \; \end{array}$$

and difference in gyro bias with:

(22) $$\delta b=b-\hat{b}$$

### Relative orientation filter: measurement equations

#### Acceleration and magnetic field update

Each phalanx contains a 3D accelerometer that provides information of both inclination and experienced inertial acceleration. The output of an 3D accelerometer can be modeled by:

(23) $$y_{a}^{b}=R^{\text{bG}}(a^{G}-g^{G})+b_{a}^{b}+e_{a}$$

where ***a***^*G*^ and ***g***^*G*^ are the inertial and gravitational acceleration respectively, both expressed in global coordinate frame, R^*b* *G*^ is the orientation from global to body coordinates, $b_{a}^{b}$ is a slowly varying sensor bias and ***e***_*a*_ is i.d.d. white Gaussian noise. The difference in accelerations experienced by two consecutive rigid bodies provides information about the relative orientation. In a pseudo static situation (***a***^*G*^≈0) the difference in inclination can be obtained. During movements the contribution of inertial accelerations is not negligible with respect to the gravitational acceleration. However, if the rotational acceleration part is significantly smaller than the translational part, it is assumed that both bodies undergo the same acceleration. In this particular situation the 3D accelerometer pair might provide both inclination and heading information.

On the hand and finger tips a 3D magnetometer is deployed. The output $y_{m}^{b}$ can be modeled (in absence of ferromagnetic materials) as

(24) $$y_{m}^{b}=R^{\text{bG}}m^{G}+e_{m}$$

where ***m***^*G*^ is the local static magnetic field, and ***e***_*m*_ is i.i.d. white Gaussian noise. The magnetometer gives information about the heading difference between various bodies.

A general equation for both accelerometer and magnetometer vector outputs ***y***_*v* *e* *c* *t* *o* *r*_ is used as a measurement update in our filter. Consider two vectors, ***r***^*i*^ and ***r***^*j*^, which are either an accelerometer or magnetometer output, measured in frame **Ψ**_*i*_ and **Ψ**_*j*_ respectively. Now the estimate of ***r***^*j*^ given in frame *i* is determined by the estimated orientation ${\hat{R}}^{\text{ij}}$:

(25) $${\hat{r}}^{i}={\hat{R}}^{\text{ij}}r^{j}$$

The difference between the true and estimated vector should be related to the error angle *δ* ***θ*** and obtained by equation 18 and 25:

(26) $$\begin{array}{lll} \delta y_{\text{vector}} & =\;r^{i}-{\hat{r}}^{i}\;\\ & =\;{\hat{R}}^{\text{ij}}\left(I_{3}+{\left[\delta \theta^{\text{ij}}\right]}_{\times }\right)r^{j}-{\hat{R}}^{\text{ij}}r^{j}+e_{r}\; & \;\\ & =\;-{\hat{R}}^{\text{ij}}{\left[r^{j}\right]}_{\times }\delta \theta^{\text{ij}}+e_{r}\; & \; \end{array}$$

Where ***e***_*r*_ is i.i.d. white Gaussian noise of the particular vector measurement.

As being mentioned before, two assumptions according the accelerometer and magnetometer measurements are made: 

1. Only inertial accelerations due to translational movements are to be expected

2. The local static magnetic field is homogeneous throughout the whole hand

Obviously these conditions are easily violated during daily life tasks and would therefore deteriorate the kinematic estimates. It is therefore necessary that the validity of relevant signals is tested before being handled by the Kalman filter. A decision algorithm is used that either accepts or rejects accelerometer or magnetometer measurements using the following conditions: 

•The absolute difference in magnitude of both the magnetometer and accelerometer output pairs is approximately equal:

(27) $$\left|{\left|\left|y_{\left\{a,m\right\}}^{i}\right|\right|}_{2}\right)-\left|{\left|\left(y_{\left\{a,m\right\}}^{j}\right|\right|}_{2}\right|<\varepsilon_{a}$$

•Accelerometer information is only accepted if the angular acceleration is negligible compared to the linear acceleration. This can be tested by the absolute angular velocities of both consecutive bodies which should be significantly small:

(28) $${\left|\left|y_{\Omega }^{\left\{i,j\right\}}\right|\right|}_{2}<\varepsilon_{b}$$

Where *ε*_*a*,*b*_ are chosen according the desired movement complexity. In addition, the measurement covariance of ***e***_*r*_ can modified each iteration step such that it scales with the error of above equations.

#### Joint dimensionality constraint

In addition to sensory input measurements, biomechanical model constraints imposed by the morphology of the hand are enforced by considering the joint constraint as an artificial measurement. The limited degrees of freedom of phalangeal joints highly constrains the relative orientation between segments. In general pronation-supination is not permitted within all phalangeal joints. In addition, only the MCP joint allows abduction-adduction. The pronation-supination angle (*γ*_*M* *C* *P*_) can be described as the angle between the *z*-axis of the hand and *x*-axis of the proximal finger frame minus 90 degrees, which can be modeled as [17]:

(29) $$\gamma_{\text{MCP}}=u_{H_{z}}^{P}\cdot u_{P_{x}}^{H}+e_{\gamma }$$

where · is the inner product operator, *e*_*γ*_ is i.i.d. white Gaussian measurement noise, and ***u*** denotes a unit vector which is projected onto a second body frame:

(30) $$\begin{array}{lll} u_{H_{z}}^{P} & ={\hat{R}}^{\text{PH}}\left(I_{3}-{\left[\delta \theta^{\text{HP}}\right]}_{\times }\right)u_{z},\;u_{z}={\left[\begin{array}{ccc} 0 & 0 & 1 \end{array}\right]}^{T}\;\\ u_{P_{x}}^{H} & ={\hat{R}}^{\text{HP}}\left(I_{3}+{\left[\delta \theta^{\text{HP}}\right]}_{\times }\right)u_{x},\;u_{x}={\left[\begin{array}{ccc} 1 & 0 & 0 \end{array}\right]}^{T}\; & \; \end{array}$$

Neglecting the error product terms, and assuming that ${\hat{\gamma }}_{\text{MCP}}=0$, yields the estimated error pronation-supination angle (*δγ*) as function of estimated error angles (*δ* ***θ***):

(31) $$\begin{array}{lll} \delta y_{\text{biomech}} & =\;\gamma_{\text{MCP}}-{\hat{\gamma }}_{\text{MCP}}\;\\ & =\;\left(u_{H_{z}}^{P}\cdot u_{P_{x}}^{H}\right)-\left({û}_{H_{z}}^{P}\cdot {û}_{P_{x}}^{H}\right)+e_{\gamma }\; & \;\\ & \approx \;-{\left[{\hat{R}}^{\text{PH}}u_{z}\right]}^{T}{\hat{R}}^{\text{HP}}{\left[\delta \theta \right]}_{\times }u_{x}\; & \;\\ & \;\;+{\left[{\hat{R}}^{\text{PH}}{\left[\delta \theta \right]}_{\times }u_{z}\right]}^{T}{\hat{R}}^{\text{HP}}u_{x}+e_{\gamma }\; & \;\\ & \approx \;u_{z}^{T}{\hat{R}}^{\text{HP}}{\hat{R}}^{\text{HP}}{\left[u_{x}\right]}_{\times }\delta \theta \; & \;\\ & \;-{\left[{\left[u_{z}\right]}_{\times }\delta \theta \right]}^{T}{\hat{R}}^{\text{HP}}{\hat{R}}^{\text{HP}}u_{x}+e_{\gamma }\; & \;\\ & \approx \;u_{z}^{T}{\hat{R}}^{\text{HP}}{\hat{R}}^{\text{HP}}{\left[u_{x}\right]}_{\times }\delta \theta \; & \;\\ & \;\;-u_{x}^{T}{\hat{R}}^{\text{PH}}{\hat{R}}^{\text{PH}}{\left[u_{z}\right]}_{\times }\delta \theta +e_{\gamma }\; & \;\\ & \approx \;\left(u_{H_{z}}^{P}{\hat{R}}^{\text{HP}}{\left[u_{x}\right]}_{\times }-u_{P_{x}}^{H}{\hat{R}}^{\text{PH}}{\left[u_{z}\right]}_{\times }\right)\delta \theta +e_{\gamma }\; & \; \end{array}$$

The finger’s PIP, DIP and thumb’s Inter Phalangeal (IP) joint constraints can be modeled in a similar fashion, only now the abduction-adduction angle *β* is constrained as well:

(32) $$\begin{array}{lll} \beta_{\text{PIP}} & =u_{P_{y}}^{M}\cdot u_{M_{z}}^{P}+e_{\beta }\;;\;\gamma_{\text{PIP}}=e_{P_{z}}^{M}\cdot u_{M_{z}}^{P}+e_{\gamma }\;\\ \beta_{\text{DIP}} & =e_{M_{y}}^{D}\cdot u_{D_{x}}^{M}+e_{\beta }\;;\;\gamma_{\text{DIP}}=e_{M_{z}}^{D}\cdot u_{D_{x}}^{M}+e_{\gamma }\; & \; \end{array}$$

### Filter design: absolute hand filter

The absolute hand orientation is calculated in a similar fashion as the relative orientations except that the orientation of the hand is calculated with respect to the global navigation frame **Ψ**_*G*_, see Figure 1.

An additional Kalman filter derived from Roetenberg *et al*. [8] is exploited to estimate the optimal absolute hand kinematics. The filter state ($\left[\begin{array}{ccc} p & v & \delta \theta \end{array}\right]$) equations are given by:

(33) $$\begin{array}{lll} {\dot{p}}^{G} & =\;v^{G}\;\\ {\dot{v}}^{G} & =\;R^{\text{GH}}a^{H}+g^{G}\; & \;\\ \delta {\dot{\theta }}^{\text{GH}} & =\;-{\left[{\hat{\omega }}_{\text{GH}}^{H}\right]}_{\times }\delta \theta^{\text{GH}}-\delta b_{\Omega }\; & \; \end{array}$$

where ***a***^*H*^ is given by equation 23 after rotation from sensor frame **Ψ**_*S* *H*_ to hand frame **Ψ**_*H*_. The position ***p***^*G*^ and velocity ***v***^*G*^ are obtained by strapdown integration [19], and $\omega_{\text{GH}}^{H}$ is the angular velocity of the hand with respect to global frame expressed in hand coordinate frame.

Orientation drift is prevented by applying a measurement update using the accelerometer and magnetometer pair positioned on the hand, where the sensor output are projected onto the gravitational acceleration and earth magnetic field respectively.

Without additional position information, integration drifts immediately occurs. Therefore, the position estimate is only acceptable for a couple of seconds. In order to reduce drifting errors, an additional zero velocity detector is implemented to detect no-movement situations in which the integrators are set to zero [20].

### Experimental methods

**Figure 3:**
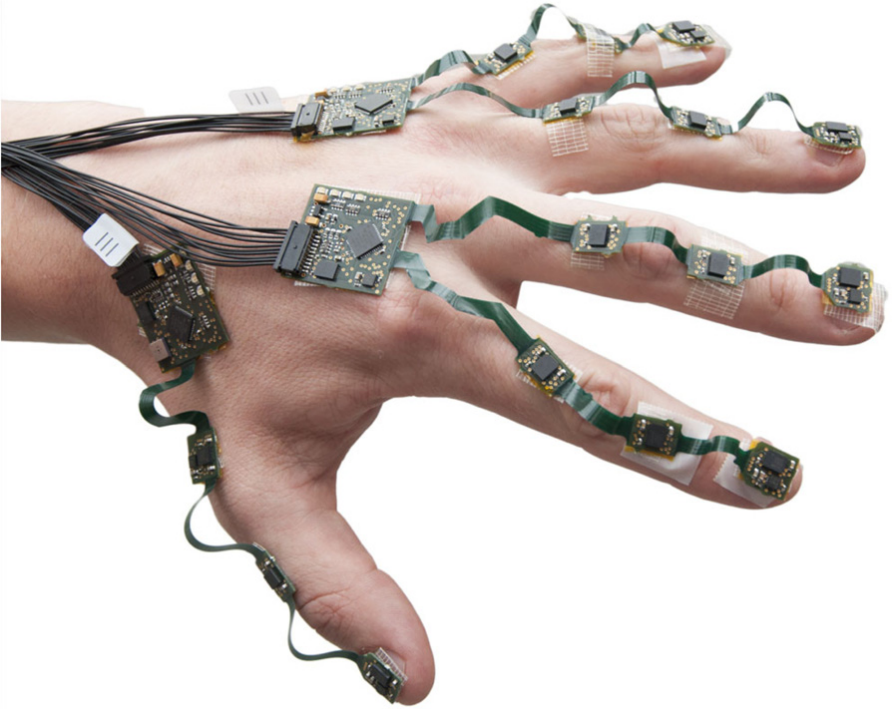
**Glove hardware consisting of multiple printed circuit board strings which are attached to each finger segment.** Every segment contains a triaxial gyroscope accelerometer pair which are connected using a flexible PCB structure. In addition, the finger tips and the back of the hand contain a triaxial magnetometer.

The instrumentation hardware of the experimental setup is depicted in Figure 3. The system contains multiple strings of flexible-rigid printed circuit board (PCB) which are mounted on the dorsal side of hand, fingers and thumb using double sided adhesive tape (experiment 1, 2, 3) or mounted on an polyamide/elastane fabricated glove (Falke) (experiment 4, 5). Each string deploys three triaxial gyroscope and accelerometer pairs (ST LSM330DLC), one for each finger or thumb segment. In addition, a triaxial magnetometer (Honeywell HMC5983) is placed on the finger’s tip and on the back of the hand. Sensor data is sampled (*@*200 Hz for the gyroscope and *@*100 Hz for the accelerometer and magnetometer) by a microcontroller (Atmel XMEGA), collected by a master microcontroller (Atmel XMEGA) and subsequently transmitted via USB to the computer. Online sensor acquisition and filter execution is performed using MATLAB^®;^. Parameters of process and sensor noise distributions is given in Table 1.

**Table 1:** Standard deviation values of accelerometer, magnetometer, angular rate, gyro bias and biomechanical constraint noise

	***e***_***a***_	***e***_***m***_	***e***_**Ω**_	***e***_***b***_	***e***_***γ***_	***e***_***β***_
	**(m/*s***^**2**^**)**	**(mG)**	**(rad/s)**	**(rad/s)**	**(deg)**	**(deg)**
*σ*	4∗10^−2^	10^−3^	5∗10^−4^	10^−4^	10^−5^	10^−5^

Given values are determined emperically using allan variances or obtained from datasheets.

The medical ethics committee acknowledged that medical ethical approval was not required for all experiments described in the next subsections, because the intent of this study is assessment of the proposed system and not the subject’s task performance.

The following subsections describe, firstly the sensor to segment calibration, next, a study with an optical reference system with one subject (experiments 1, 2), subsequently, a second study with one subject without optical reference (experiments 3, 4), and finally in a third study the repeatability of the system which was performed by 5 subjects. It should be noted that in all experiment the subject was seated at a table.

#### Sensor-to-segment calibration

Assessment of hand and finger kinematics, and thus all described experiments, requires a mapping from sensor coordinate frames to the corresponding segment frames. In this typical calibration problem we seek for the transformation matrix from sensor frame to segment body frame. First a coordinate frame should be defined within the particular segment of the hand, which is given in the first methods section. Next, after attachment of the sensor PCB to the finger segment, one should determine the orientation between both frames, for example between **Ψ**_*S* *H*_ and **Ψ**_*H*_, see Figure 1.

In order to construct this relative orientation, subjects were asked to perform the following procedure: 

1. Place the hand on a flat surface with the back of the palm pointing up. This defines the abduction-adduction axis (*x*), given by the accelerometer output:$e_{x}^{\text{Seg}}=\frac{y_{a}}{{\left|\left|y_{a}\right|\right|}_{2}}$

2. Raise the hand and flex the MCP, PIP and DIP joint of all fingers. During movement avoid abduction and adduction of the MCP joint. This defines the flexion-extension axis given by the gyroscope output: $e_{z}^{\text{Seg}}=\frac{y_{\Omega }}{{\left|\left|y_{\Omega }\right|\right|}_{2}}$.

The sensor to segment orientation is given by:

(34) $$R^{\text{SegSen}}=\left[\begin{array}{lll} e_{x}^{\text{Seg}} & \left(e_{z}^{\text{Seg}}\times e_{x}^{\text{Seg}}\right) & e_{z}^{\text{Seg}} \end{array}\right]$$

The phalangeal segment lengths ***p***_*i* *j*_ were approximated by first palpation of various joints and subsequently measuring the positions using a ruler. Alternatively or as a first guess, segmental lengths can be estimated using a regression model of the hand and a measure of hand width and length [21,22].

#### Finger tip position comparison relative to an optical system

In the first experiment the accuracy in terms of finger tip position of one subject was analysed. Two tests were performed, in which the the subject started and finished with their hand flat on a table top and repeated each movement sequence 10 times with an interval of 1 minute.

In the first test, the subject was asked to repeatedly flex the index finger up to maximum flexion angle of MCP, PIP and DIP joint respectively, while having the arm horizontally stretched such that the back of the hand’s palm is directed upwards. The cyclical movement has to be performed five times with a time period of approximately 1 second.

In a second test, the subject made circular-like movements with an stretched index finger while the hand maintained a static posture such that the ab-adduction angle of the MCP joint was maximised each repetition. Similar to the first test, the cyclical movement was performed five times with an period time of approximately one second.

**Figure 4:**
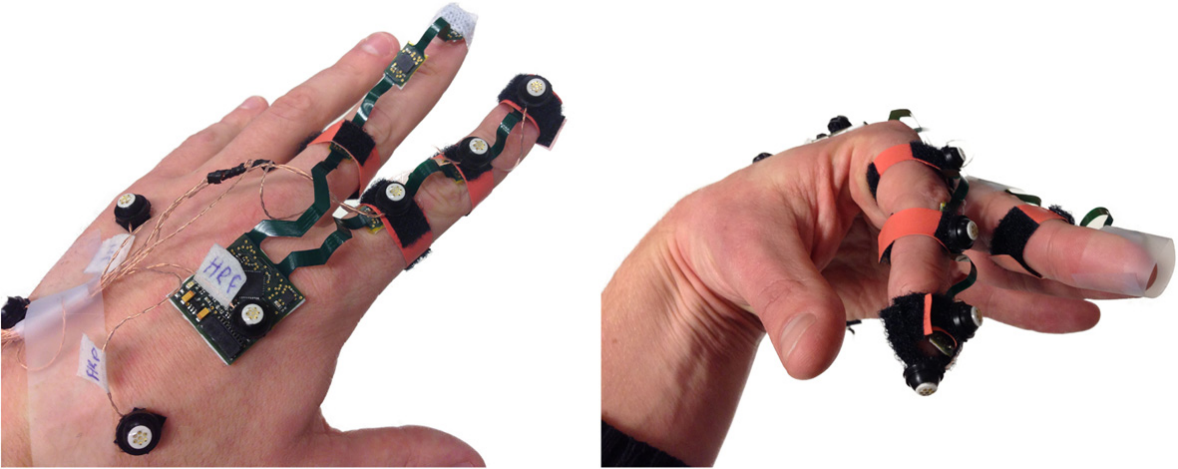
**Hand and index finger instrumented with both inertial sensors and active optical markers.** Four markers on the back of the hand define the hand coordinate frame.

The estimated finger tip position was compared to the measurement output of an optical tracking system VZ-4000 (PTI VisualEyez). Both hand and index finger were instrumented with active optical markers placed on top of the inertial sensors, see Figure 4. An additional marker was placed on the index finger’s tip. The position of tip is expressed in a common hand coordinate frame defined by the hand markers and aligned with the hand frame of the inertial sensor placed on the hand.

#### Index finger and thumb pinching motion

During the second experiment a subject was asked to perform cyclical pinching movements of the thumb and index finger in which the tips of index finger and thumb touched each other at the end of the pinching movement. A spot was drawn on both tips and the subject was asked to coincide the spots during pinching as accurate as possible. The movement was performed 10 times in which each trial contains 10 cyclical pinching motions. The subject kept contact during the touching phase for about one second in which the hand could be oriented in any direction. During the touch phase, the position of both index finger and thumb tip was estimated.

#### Dynamic range

In order to illustrate the ability to keep track of rapid finger movements a test was performed in which 30 repeated flexion-extension movements with all fingers joints were made. The subject was asked to start with the hand flat on a tabletop, raise the forearm and perform full hand opening and closing movements according a metronome tempo of 116 BPM. This is the highest rate that can be achieved before clipping of the gyroscope signal (2000 deg/s) occurs. The PIP angle of the index finger was evaluated during the repeated flexion-extension movement. It was asked to keep the thumb joints extended such that the range of the PIP joint is constrained by the subject’s minimum and maximum PIP flexion angle.

#### Repeatability

The reliability of the system was assessed by determining the repeatability of the finger joint orientations in a defined hand posture. A standardized dataglove evaluation protocol, proposed by Williams et al [23] and frequently applied to evaluate datagloves [4,24] was partly adopted. The protocol includes a gripped and flat hand position test respectively. For both tests Willams distinguished between with and without donning-doffing between the measurements. The donning-doffing tests were excluded in our study as the current hardware is not integrated in textile and the non donning-doffing tests mimic the proposed applications sufficiently over a short and long measurement duration.

Five healthy male volunteers, aged 21-53 years, with no known hand disorders participated in this experiment. All tests were performed by the subject’s left hand, and depending on the size of the hand, either a small or large instrumentation set was fastened. The difference between a small or large instrumentation set lies only in the length of the flexible PCB structure.

In the first test subjects placed their flat hand on a table top within a designated area. This area is indicated by the contours of the subject’s hand and used as a guideline to securely maintain hand position during the flat phase. Then the subject was asked to raise his hand, flex all finger and thumb joints, maintain this posture for 6 seconds and finally return back to the flat phase position for 6 seconds. This flexion/flat cycle was repeated 10 times.

The second test comprised a hand posture where a plaster mold was clenched. Prior to start of the experiment the subject moulded a heated thermoplastic material (ProtoPlast®;) which returned to a solid state upon cooling.

During the experiment the participant clenched the mold for 6 seconds and subsequently released for 6 seconds. This clench/release cycle was repeated 10 times. Subjects were allowed to maintain the hand in any orientation during the clench phase.

For both tests the subject was asked to repeat the measurement 10 times, where a pause of 1 minute was included between each measurement.

The repeatability of both tests is indicated by the range and standard deviation (SD) of all joint angles over all trials and subjects during the flat phase (test 1) and during the clench phase (test 2).

## Results

### Finger tip position comparison

As mentioned in the Methods section, two movement conditions were performed and compared to an optical tracking system. Prior to start of the experiments a required calibration trial was conducted to firstly align the hand coordinate frames of optical and inertial sensors, secondly to obtain the position of the MCP joint expressed in the hand coordinate frame and finally to obtain the position of the tip LED expressed in the distal coordinate frame.

**Figure 5:**
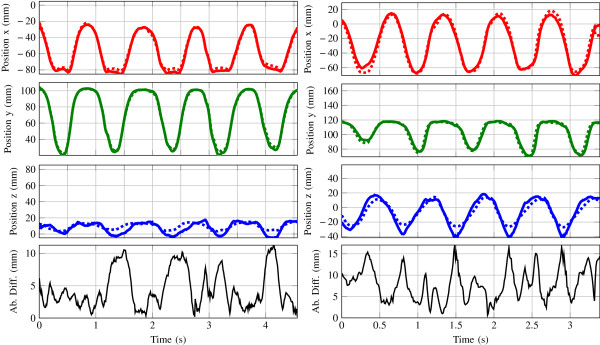
**Reconstruction of the index finger position (*x*,*y*,*z*) during a flexion-extension (left) and circle drawing movement (right) measured by the optical system (solid) and estimated by the inertial sensors (dashed).** The lower plots show the absolute index finger tip position difference.

On the left, Figure 5 shows the position of the finger tip with respect to the back of the hand for a representative trial of the first experiment where the index finger was flexed and extended repeatedly. The error in tip position is defined as the absolute distance difference between estimated tip position by our inertial sensor system and optically measured tip position. It can be seen that the largest error contribution is caused by an error in the *z*-direction during maximum flexion (up to 10 mm).

On the right, Figure 5 shows a representative trial for the second movement type where circle shaped movements were performed with the index finger. A large error is mainly visible in the minima of the *z*-direction which corresponds with the maximum abduction angle of the MCP joint.

Table 2 shows the RMS differences between both measurement systems of both movement types. Mean and standard deviation values over 10 trials is given. The finger tip position correspondence was within 5.0±0.5 mm for index finger during flexion movements and 12.4±3.0 mm during circle shaped movements. For both movement types the largest contribution of the total error was due to the difference in the *z*-direction. This is presumably caused by a misalignment of both sensor coordinate frames and the accumulation of errors caused by joint model imperfections and an incorrect estimation of segment lengths.

**Table 2:** RMS differences in estimated (inertial) and measured (optical) index finger tip position

	**Flexion Extension**			**Circles**	
	**Mean (mm)**	**std. dev. (mm)**		**Mean (mm)**	**std. dev. (mm)**
***p***_*d*,*x*_	2.2	0.4		5.7	2.2
***p***_*d*,*y*_	2.0	0.5		4.9	1.3
***p***_*d*,*z*_	3.3	0.6		9.7	2.1
|| ***p***_*d*_||	5.0	0.5		12.4	3.0

Both movement types (Flexion/ Extension and Circle-like) were repeated 10 times.

### Index finger and thumb pinching motion

In the third, so called pinching test, the distance difference was calculated during each contact phase of the thumb and index finger tips.

In order to minimise reconstruction errors due to the rather coarse ball-socket model used for the thumb’s CMC joint, the subject is instructed to avoid rotations of this joint as much as possible. However, this constraint did not hinder the ease of movement. The average RMS distance difference over all pinched movements is 6.5±2.1 mm which is 3.7±1.2*%* of the maximum tip distance difference, obtained during the maximum extension phases.

The average distance displacement of index finger and thumb was 65.6±2.2 mm and 15.7±2.5 mm respectively.

### Dynamic range

**Figure 6:**
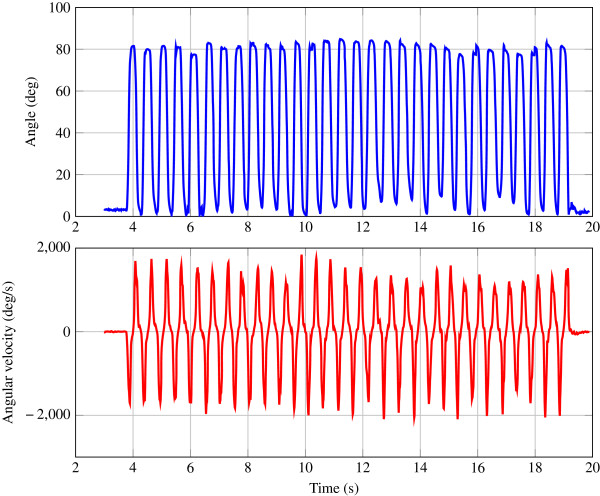
Helical angles (upper) and corresponding angular velocity (lower) of the index’ PIP joint during 30 repeated flexion-extension movements of one subject.

A representative trial of a rapid flexion motion of the index finger is depicted in Figure 6. Shown are the estimated PIP angle along with the corresponding angular velocities of the index finger.

As can be seen, the total transition time was approximately 0.5 s for a full extension-flexion-extension cycle corresponding to the 116 BMP rate of the metronome. The difference in helical angles between minimum and maximum flexion was about 80 degrees, being determined in an independed static trial. Please note that the range of approximately 80 degrees is reconstructed each cycle with the derivative of the angular velocity approaching zero before the movement changes directions in several of the cycles, while the angular velocity stays within the range of the gyrosopce (+/− 2000 deg/s). The bandwidth of the filter is apparently large enough to track these fast movements through the whole range.

### Repeatability

**Figure 7:**
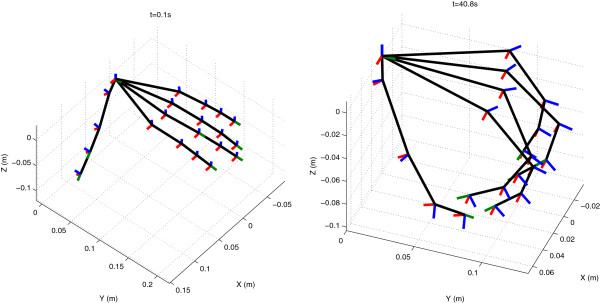
Representative hand posture reconstruction for both flat (left) and flexed fingers (right).

Figure 7 shows two representative reconstructions of one subject during the repeatability tests. On the left figure the flat hand phase is depicted, whereas on the right the flexion phase is depicted. Table 3 shows the average range, which is the mean difference between maximum and minimum angle of each trial, and standard deviation of all joint angles during the flat hand phase (test 1) and during the clenching phase (test 2) for each subject. In addition, mean range and standard deviation values over all subjects is listed (1.1±0.4 deg) and (1.8±0.6 deg) as well as the mean values obtained from other studies with different data gloves.

**Table 3:** Results of repeatability analysis

**Subject**	**Flat hand**		**Custom plaster mold**	
	**Range**	**std. dev.**	**Range**	**std. dev.**
	**(deg)**	**(deg)**	**(deg)**	**(deg)**
1	1.4	0.5	1.9	0.6
2	0.7	0.2	1.9	0.6
3	1.2	0.4	1.8	0.6
4	0.9	0.3	1.8	0.6
5	1.3	0.4	1.7	0.5
Mean value	1.1	0.4	1.8	0.6
Dataglove (Wise et al. [23])	4.5	1.6	6.5	2.6
Humanglove (Dipietro et al. [4])	3.8	1.2	7.5	2.4
Shadow monitor (Simone et al. [25])	1.5	0.5	5.2	1.6
WU glove (Gentner et al. [26])	2.6	0.9	6.1	1.9

Mean range (difference between maximum and minimum angle during one trial) and standard deviation is given for all joint angles of each subject during the hand flat phase and during clenching of a custom plaster mold. In addition, mean values of this study and mean values from others studies are given.

## Discussion

Small scaled inertial and magnetic sensors combined with a biomechanical model of the hand, embodied in an Extended Kalman Filter framework, result in a promising tool to assess 3D kinematics of the human hand in a quantitative manner.

In addition to existing glove systems which are often restricted to measuring a maximum of 2 DoF per joint, full 3D angles can be measured. This allows, for example, assessment of pronation-supination of various MCP joints.

To our knowledge existing glove systems have only been validated in static situations using repeatability tests described by Williams et al [23]. Assessment of actual tip positions and dynamic range have never been examined but are of great importance when the glove is to be used for daily-life manipulation tasks and therefore have been evaluated as well.

We have observed that the estimation accuracy strongly depends on the sensor to segment calibration procedure. In contrast to our approach, most of the existing data gloves measure across the joints and therefore give an output which is a direct function of the joint’s angle. Calibrating such gloves might be easier but also tend to be more error-prone after long utilisation periods, as the sensor’s axis should be aligned perfectly with the anthropomorphic joint axis.

The current system requires palpation measurements to determine phalangeal segment lengths and position of MCP joints centres with respect to a common hand reference frame. Accurate determination of functional joint position (MCP) and the axis of rotation (PIP, DIP) would improve the accuracy and decrease time and effort needed for sensor to segment calibration. The current filter can be extended such that those parameters are included and estimated online. In addition, the dominant functional segment axes are found by performing dedicated movements. However, if a certain neuro-muscular patient group may not be able to perform such movements, customised sensor to segment calibration procedures may be required.

Likewise, the optical reference system requires a proper sensor to segment calibration. Independent segment orientation measurements require at least three rigidly connected markers attached to each finger segment. With a specified position accuracy of 0.5 mm RMS, markers should be separated sufficiently (>6 cm) to obtain an orientation accuracy less than 1 degree. Hence, this is almost impossible to accomplish for each finger segment due to either mounting space, occlusion difficulties or limitation of movement freedom. Therefore, we chose to compare finger tip positions calculated by both systems. However, this approach has a limitation because the error introduced in either the estimated orientation or measured segment length accumulates to an error in the estimated finger tip position, which makes localisation of the exact error source much more challenging. As the largest uncertainty is caused by determination of segment origins we expect that the primary contribution to the position difference is due to misalignments of both measurement systems.

All joints are considered to be perfect ball-socket hinges with 3 DoF. Joint dimensionality is soft-constrained by adding uncertainty to non-natural rotation axes which allows some joint laxity and imperfections. It should be noted that the soft-constrained updates assume that the rotational axes of multi DoF joint are intersecting and orthogonally directed. However, these assumptions are not valid and results in erroneous reconstructions [22]. This for example visible in Figure 5, where the reconstruction of the index finger position is much worse when the ab-adduction angle is maximal. This could be caused by disregarded pro-suppination rotations, non-orthononal rotation axes, or a translation of the joint’s origin. Likewise for the thumb’s carpo-metacarpal joint (CMC) a perfect ball socket joint is considered, which is obviously not the case. Mitigating those errors demands improved, and more complex, biomechanical joint models [22,27]. Adaption of such models is the next step to improve the overall performance.

The described method uses six Kalman filters, one for each finger and one for the hand. This decoupled approach neglects some existing synergies within the hand, which means that not all available and relevant information is used in the model. However, it keeps the filter manageable in terms of computational resources, tuning and prevents unwanted coupling between states due to modeling errors. Alternatively, one could choose for a centralized structure, with one large state vector. This approach allows inclusion of synergies and therefore improve the kinematic estimates. However, the state will have approximately 100 elements and might require alternative, more sophisticated, filtering methods.

The current filter assumes a perfect homogeneous magnetic field throughout the hand. When interacting with ferro-magnetic object this assumption is easily violated. It is therefore necessary to extend the filter such that local magnetic field disturbances are tracked during operation and don’t affect the estimated kinematics. Nevertheless the magnetic field is only required for observability in long static posture periods.

For this study sensors were both mounted directly on the subject’s skin as well as on a glove fabricated by an elastic textile. Movement artefacts caused by skin and textile deformations have not been investigated thoroughly. We expect that those artefacts are negligible with respect to errors caused by imperfection of the biomechanical joint model. Moreover, since the applied PCB’s are light-weighted and have a flexible structure, visual inspection and feedback from the subjects confirmed that the sensors can be worn unobtrusively without hampering hand and finger movements. Nevertheless, a donning-doffing study should be performed whenever a suitable textile has been selected wherein the sensors can be integrated.

Our repeatability study showed similar (flat hand test) to slightly better (mold test) results compared to existing studies of different dataglove systems. It should be mentioned that those studies only evaluated the sensor variability over time. Simone et al [25], evaluated the “Shadow Monitor” which has a simpler approach and showed a comparable reliability. However, only a reduced set of joint angles could be measured. This is in contrast to our approach where all joint angles were estimated and evaluated using custom filters.

Although the repeatability studies are promising, additional testing of the absolute accuracy using an independed reference system is necessary. These studies should include multiple subjects in which all joint angles should be evaluated, especially during arm movements in which the hand is oriented in different poses.

It is shown in various studies that the accuracy of orientation estimates using inertial sensors is higher than joint orientation estimation shown in this study [14-16]. Hence, the tracking accuracy of hand kinematics is most probably not limited by inertial sensor accuracy nor the Kalman filter approach.

## Conclusion

The first results of our sensing system are favourable compared to existing datagloves. It is able to adequately reconstruct finger tip movements (<13 mm), high dynamic range (116 full range finger movements per minute) and adequate repeatability (<2 degrees). This makes the inertial sensor approach promising, especially, when bearing in mind that consumer-grade inertial sensors are getting smaller and less expensive, whereas the quality vastly improves. This study showed the possibilities and challenges to be faced when inertial sensing technology is applied for kinematic analysis of the human hand and fingers.

## Appendix

### Derivation error angle dynamics

Derivative of the parameterized orientation:

(35) $$\dot{q}=\dot{\hat{q}}⊙\delta q+\hat{q}⊙\dot{\delta q}$$

gives:

(36) $$\frac{1}{2}q⊙\omega =\frac{1}{2}\hat{q}⊙\hat{\omega }⊙\delta q+\hat{q}⊙\dot{\delta q}$$

(37) $$\hat{q}⊙\dot{\delta q}=\frac{1}{2}q⊙\omega -\frac{1}{2}\hat{q}⊙\hat{\omega }⊙\delta q$$

(38) $$\begin{array}{lll} 2\dot{\delta q}={\left[\begin{array}{ll} 0 & \dot{\delta \theta } \end{array}\right]}^{T} & =\;\left(\delta q⊙\omega -\hat{\omega }⊙\delta q\right)\;\\ & =\;\Omega (\omega )\delta q-\Gamma (\hat{\omega })\delta q\; & \;\\ & =\;\left[\begin{array}{cl} 0 & -{\left(\omega -\hat{\omega }\right)}^{T}\\ \left(\omega -\hat{\omega }\right) & -{\left[\omega +\hat{\omega }\right]}_{\times } \end{array}\right]\;\left[\begin{array}{l} 1\\ \frac{1}{2}\delta \theta \end{array}\right]\; & \; \end{array}$$

Where Ω(***ω***) and $\Gamma (\hat{\omega })$ are the left and right quaternion product operators [12]. Subsequently, by taking the second row and neglecting second order terms gives:

(39) $$\dot{\delta \theta }=-{\left[\hat{\omega }\right]}_{\times }\delta \theta +\delta \omega$$

with:

(40) $$\delta \omega =\omega -\hat{\omega }$$

Now we can calculate the dynamics originating from angular velocity differences measure:

(41) $$\begin{array}{lll} \;\delta \omega_{\text{ij}}^{j} & =\omega_{\text{ij}}^{j}-{\hat{\omega }}_{\text{ij}}^{j}\;\\ & =\left[\left(y_{\Omega }^{j}-b_{\Omega }^{j}-e_{\Omega }^{j}\right)-R^{\text{ji}}\left(y_{\Omega }^{i}-b_{\Omega }^{i}-e_{\Omega }^{i}\right)\right]\; & \;\\ & \;-\left[\left(y_{\Omega }^{j}-{\hat{b}}_{\Omega }^{j}\right)-{\hat{R}}^{\text{ji}}\left(y_{\Omega }^{i}-{\hat{b}}_{\Omega }^{i}\right)\right]\; & \;\\ & =-\delta b_{\Omega }^{j}-e_{\Omega }^{j}+R^{\text{ji}}e_{\Omega }^{i}-{\hat{R}}^{\text{ji}}\left[I_{3}+{\left[\delta \theta^{\text{ji}}\right]}_{\times }\right]\left(y_{\Omega }^{i}-b_{\Omega }^{i}\right)\; & \;\\ & \;+{\hat{R}}^{\text{ji}}\left(y_{\Omega }^{i}-{\hat{b}}_{\Omega }^{i}\right)\; & \;\\ & =-\delta b_{\Omega }^{j}-e_{\Omega }^{j}+R^{\text{ji}}e_{\Omega }^{i}-{\hat{R}}^{\text{ji}}\;{\left[\delta \theta^{\text{ji}}\right]}_{\times }\;\left[y_{\Omega }^{i}\;-\;b_{\Omega }^{i}\right]\;+\;{\hat{R}}^{\text{ji}}\delta b_{\Omega }^{i}\; & \;\\ & ={\hat{R}}^{\text{ji}}{\left[y_{\Omega }^{i}-b_{\Omega }^{i}\right]}_{\times }\delta \theta^{\text{ji}}+{\hat{R}}^{\text{ji}}\delta b_{\Omega }^{i}-\delta b_{\Omega }^{j}-e_{\Omega }^{j}+R^{\text{ji}}e_{\Omega }^{i}\; & \; \end{array}$$

Non stationary gyro bias can be modeled by a random walk process:

(42) $$\dot{b_{\Omega }}=e_{b}$$

The error bias process is given by:

(43) $$\begin{array}{lll} \dot{\delta b_{\Omega }} & =\;\dot{b_{\Omega }}-{\dot{\hat{b}}}_{\Omega }\;\\ & =\;e_{b}\; & \; \end{array}$$

### Quaternion mathematics

#### Rotation matrix

(44) $$R(q)=qq^{T}+q_{0}^{2}I_{3}+2q_{0}{\left[q\right]}_{\times }+{\left[q\right]}_{\times }^{2}$$

where ***q*** and *q*_0_ are the vector and scalar quaternion part.

#### Quaternion exponential

(45) $$\text{exp}(\theta )=\left[\cos{\left|\left|\theta \right|\right|}_{2},\frac{\theta }{{\left|\left|\theta \right|\right|}_{2}}\sin{\left|\left|\theta \right|\right|}_{2}\right]$$

## Competing interests

The authors declare that they have no competing interests.

## Authors’ contributions

HK developed the kinematics algorithms, prepared and conducted the experiments and drafted the paper. VS developed the hardware. DR and PV contributed in filter design and drafting the paper. PV is leading the PowerSensor project. All authors read and approved the final manuscript.
